# A Medical License for Ladies

**Published:** 1874-03

**Authors:** 


A Medical License For Ladies.—The King and Queen's College of Physicians in Ireland has determined to admit females to the examination for its diploma in Midwifery, which can be registered. The Royal College of Surgeons in England has the power of granting a license in Midwifery, which can be registered. If it would use this power in favor of women passing a fit examination, we might hope for some abatement of the scandal of midwives' midwifery, and see an experiment on a goodly scale of the fitness of women tor midwifery practice, which we much question.—*The Lancet.*

